# Supporting Parents of Youth with Chronic Pain: A Mixed Methods Evaluation of a Supportive Educational Intervention

**DOI:** 10.3390/children13010063

**Published:** 2025-12-31

**Authors:** Megan Mackenzie Sweeney, Samantha Levy, Alisha Jean-Denis, Lonnie Zeltzer, Tori R. Van Dyk

**Affiliations:** 1 Department of Psychology, School of Behavioral Health, Loma Linda University, 11130 Anderson Street, Loma Linda, CA 92350, USA; 2 Creative Healing for Youth in Pain, 5041 Valjean Avenue, Encino, CA 91436, USA

**Keywords:** pediatric chronic pain, parenting program, adolescent, group intervention

## Abstract

**Highlights:**

**What are the main findings?**

- Creating Bonds, an 8-week program for parents of youth with chronic pain, was effective and beneficial in improving parents’ knowledge, coping, and emotional wellbeing.
- Collectively, parents described the program as educational, validating, and transformative, emphasizing the dual benefit of actionable tools and psychosocial support.

**What are the implications of the main findings?**

- Parent-focused interventions are a critical and effective component of pediatric chronic pain care.
- Virtual parent programs are feasible, scalable, and capable of reducing barriers to care.

**Abstract:**

**Background**: Parents of youth with chronic health conditions face several challenges in supporting their children across contexts. Involvement of parents in a child’s pain management approach is accepted as best practice, yet there is little guidance on how to best parent the child with chronic pain. Prior studies have shown that parents require support and education to effectively care for their children and themselves. This quality improvement program evaluation aimed to evaluate group-level: (1) feasibility of the Creating Bonds program, (2) acceptability and perceived effectiveness of the program, and (3) suggestions for program improvements. **Methods**: In this quality improvement program evaluation, parents (*N* = 40) of youth with chronic pain from the United States and Europe were recruited online to participate in a virtual peer-support and educational program, Creating Bonds, offered through the nonprofit organization, Creative Healing for Youth in Pain. Creating Bonds is an 8-week, virtual, supportive, and educational program for parents and caregivers of youth with chronic pain led by a licensed clinical psychologist. A mixed methods approach evaluated the impact of and suggestions for improving the program. Independent samples *t*-tests were used to examine quantitative items related to understanding of pain, isolation, confusion, distress, relationships, and self-care. Qualitative responses were evaluated for common themes through an inductive thematic analysis. **Results**: Results indicated that Creating Bonds significantly improved parents’ level of understanding of chronic pain, relationships with others, and self-care, and significantly reduced confusion about parenting a child with chronic pain, stress, and anxiety levels (*p*s < 0.05). Levels of isolation moderately decreased. Parents qualitatively described the experience as validating, connecting, and educational, with both emotional relief and practical strategies emerging as benefits. **Conclusions**: Quantitative results and qualitative themes capture the dual role of the Creating Bonds program in providing tangible parenting tools alongside education and critical psychosocial support. Parents entered with uncertainty, a desire for strategies, and hope for connection, and they came away with validation, practical parenting tools, and a community facing similar experiences.

## 1. Introduction

Chronic pain, defined as persistent or recurring pain that lasts three months or longer, affects over one in five children and adolescents in North America and Europe, and represents a significant global health concern [1]. Pediatric chronic pain is best understood within a biopsychosocial framework, reflecting interactions among biological, psychological, and social processes involved in pain perception and management. Pediatric chronic pain can have significant negative effects on a child’s daily life [2,3,4]. Youth with chronic pain experience substantial impairments in physical and psychological functioning, quality of life, and emotional well-being, with higher rates of anxiety and depression than peers without pain [2,3,4,5]. Adolescence is a particularly vulnerable period for pain onset, with studies showing increases in pain prevalence between 12 and 14 years of age [6]. Chronic pain can disrupt school attendance and social participation, negatively affecting development during critical life stages [3]. Despite its prevalence, pediatric chronic pain remains difficult to manage due to developmental variability, communication challenges, and the multifaceted nature of pain [7,8,9].

A child’s experience of pain and pain-related beliefs do not develop in isolation but within the family context [10]. Family systems theories emphasize the interdependence of family members, such that changes in parent functioning may influence child health outcomes [11]. Parent and family factors such as parental chronic pain, emotional distress, pain-related beliefs, and parenting behaviors are strongly associated with child pain intensity, disability, and healthcare use [12,13,14,15]. In particular, parental pain catastrophizing, characterized by rumination, magnification, and helplessness in response to a child’s pain, has been linked to greater child pain and functional impairment [16]. Parental pain catastrophizing may increase parental attention to child pain behaviors in a way that inadvertently increases the child’s pain severity, frequency, and disability [17]. Through social learning processes, children may adopt maladaptive pain beliefs and coping strategies modeled by parents, even in the absence of severe pain experiences shared by parents. Consistent with this, parental chronic pain is associated with increased risk of pain in offspring across clinical and community samples [18]. A compelling conceptual framework has recently emerged related to “chronic pain contagion” among families coping with pediatric chronic pain, outlining how ongoing suffering is jointly experienced by the child and parent, producing maladaptive changes for both the parent and child [19]. 

Research shows that parents exert their own parenting styles drawing from how they were parented, how they have seen others parent, and how they have historically cared for others [20]. Parenting a child with chronic pain presents unique challenges, as caregiving responses that are adaptive in acute pain contexts may be maladaptive when pain becomes chronic. Parenting styles marked by maternal overprotection, over-involvement, and enmeshment are common in families of youth with chronic pain and are associated with poorer functional outcomes [17,21,22]. For example, a main tenet in chronic pain is that function improves before the pain lessens, advising that children have to cope with the pain and move through it before achieving notable pain reduction. This approach often feels counterintuitive to parents because the natural response to a child’s acute pain experiences is increased caretaking and attention (e.g., frequent asking about pain, attending to pain behaviors, reducing expectation for normal functioning until pain subsides, accommodating the pain). Well-intentioned parental responses may inadvertently reinforce pain-related disability over time [23,24].

Evidence has demonstrated that parents of children with chronic pain also experience their own negative emotional, mental, and social outcomes [25]. Among families who have an adolescent with a chronic pain condition, parental distress associated with caregiving burdens may tax parents’ psychological resources (i.e., attention, patience, and warmth that support effective parent–child communication). Parents frequently report uncertainty about how to respond effectively to their child’s pain and difficulty understanding its biopsychosocial underpinnings [23,26].

Parent-focused interventions have demonstrated benefits for both parents and children with pain [27]. Parents with regular adaptive self-care practices are better able to endure chronic stress and respond to the needs of the child in pain in developmentally appropriate ways [28]. Psychological, educational, and skills-based programs targeting parental coping, self-care, and psychological flexibility have been associated with reductions in parental distress, catastrophizing, and protective parenting behaviors, as well as improvements in child functioning and pain outcomes [29,30]. Virtual group-based interventions may additionally enhance accessibility and feasibility for parents and families [30].

Despite the growing evidence that supports including parents, pediatric chronic pain treatments continue to focus primarily on the child, with limited attention devoted to parent or caregiver needs [31]. Interventions that explicitly include parents have demonstrated improvements in parental coping and emotional functioning alongside reductions in child pain and disability [32,33]. Given the high prevalence of chronic pain during adolescence, supporting parents remains a critical and underutilized component of comprehensive pediatric pain management. Interventions that address parental well-being and caregiving responses may yield downstream benefits for children’s health, functioning, and developmental outcomes.

Creating Bonds is an 8-week program for parents of youth with chronic pain that was developed in 2022 by an experienced pediatric pain psychologist. The program is part of a nonprofit organization, Creative Healing for Youth in Pain (CHYP), which aims to provide education, peer support, and creative healing experiences to families of youth and young adults with chronic pain. Prior research studies have evaluated outcomes of educational and supportive interventions for parents of youth with chronic pain as adjuncts to programs aimed at treating youth with chronic pain. However, the present feasibility and acceptability evaluation of a real-world, standalone parent program delivered virtually to parents without requiring child participation in a pain program is novel. The mixed method design aimed to evaluate the Creating Bonds program, to understand parents’ experiences in the program, and to elucidate future suggestions for improving the program. Qualitative methods are particularly valuable to supplement quantitative findings as qualitative responses provide rich, in-depth insights into personal experiences and beliefs that quantitative approaches may not fully capture [34].

This quality improvement pilot program evaluation aimed to evaluate group-level: (1) feasibility of the Creating Bonds program, (2) acceptability and perceived effectiveness of the program, and (3) suggestions for program improvements. Feasibility and acceptability of the program were examined through parent attendance and parental perceptions of the program before and after participation. It was hypothesized that the 8-week Creating Bonds program would be well-attended and accepted by parents. Effectiveness was evaluated through group-level changes in parents’ self-reported understanding of chronic pain, self-care practices, and feelings of isolation, anxiety, stress, and confusion, all of which were hypothesized to improve after program participation. Finally, qualitative feedback was evaluated to support effectiveness and suggestions for future program improvements.

## 2. Materials and Methods

Approval was obtained from the Loma Linda University Institutional Review Board, and the program evaluation for quality improvement purposes was deemed not human subjects research. All program attendees provided web-based informed consent to participate in the Creating Bonds program prior to data collection. Participation remained voluntary throughout the duration of the program. In order to keep feedback anonymous, no demographic or identifying information was collected.

### 2.1. Participants and Procedures

Parents were recruited to participate via the CHYP website (www.mychyp.org), which offered a description of the series, date and time, and an opportunity to register directly through the website. Parents were informed about the series through several methods, including healthcare providers, search engine responses to individual queries related to parenting youth with pain, and through other CHYP programs. Creating Bonds groups were offered once a week for 75 min each week, for a total of 8 weeks. Before and after the 8-week program, parents completed a quantitative questionnaire to assess understanding of pain, feelings of isolation, confusion about how to parent the child in pain, parental stress and anxiety levels, relationship challenges, and the parents’ self-perceived abilities to care for themselves. Qualitative items assessed what parents were hoping to discuss during the program, their experience in the program, what they learned, and recommendations for future program improvements.

Over a 2-year period (2023–2025), 6 sessions of Creating Bonds were offered, typically in March, May, and October of each year. Parents were eligible for the Creating Bonds Program if they were English-speaking, had access to an electronic device to participate in Zoom sessions, and had a child diagnosed with chronic pain, defined as pain for a period of at least three months, at the time of enrollment. After parents were informed about the program and dates of the series through the CHYP website, an electronic informed consent form was sent to the parent to sign and return at least 1 week before the start date of that parent’s Creating Bonds program. One week prior to the start date, parents were also sent an email asking them to complete an optional electronic pre-program questionnaire containing 7 quantitative items and 1 qualitative item. They were also provided with the weekly Zoom link and password to join the virtual Creating Bonds sessions. All parent attendees and the program instructor logged onto the program Zoom link at the same time every week for 8 weeks. After the 8-week series, parents were administered a post-program questionnaire with 8 quantitative items and 3 qualitative items. Only one parent or caregiver from each family could participate in the Creating Bonds series at a time. Due to the entirely virtual nature of the Creating Bonds Program and lack of geographic restrictions, parent attendees across the United States and Europe were able to participate.

### 2.2. Creating Bonds Program

The 8-week Creating Bonds program was led by a licensed clinical psychologist who developed the program and specialized in pediatric pain management. Over the 8 weeks with sessions occurring weekly for 75 min each session, the goal was to equip parents with education and skills to parent the child who has chronic pain, as well as to reduce isolation by creating a community of other parents facing similar challenges. The Creating Bonds program included psychoeducation, information on how the family plays a role in chronic pain management, mindfulness strategies, evidence-based parenting strategies, and other topics listed in Table 1 and Table 2. Each of the 8 sessions in Creating Bonds was informed by input from group members. The facilitator provided guidance for group introductions at the beginning of the first session and led a mindfulness exercise for the first few minutes of each session. See Table 1 for mindfulness exercises led by the group facilitator. Specific content within each session was initiated by parents participating in each group as it came up naturally.

### 2.3. Measures

#### 2.3.1. Feasibility

Each parent’s attendance was based on whether they joined the Zoom link for each weekly session. At the end of the 8-week series, the post-program questionnaire asked participants to report how many sessions they attended. Parents were sent a reminder via email with the link to join the session each week.

#### 2.3.2. Acceptability and Perceived Effectiveness

The questionnaires were developed by the program facilitator and pediatric pain researchers at CHYP. On a yes/no scale, parents were asked if they knew any parents who have children with chronic pain. Parents were also instructed to answer six quantitative items on both the pre- and post-program questionnaires on a 1–10 scale, in which 1 indicated the least amount, and 10 indicated the greatest amount. Pre- and post-program questionnaire items included, “What is your level of understanding of pediatric chronic pain?”; “How isolated do you currently feel because of having a child with pain?”; “How confused do you feel about how to parent differently than you usually would, such as when to push and when to coddle?”; “How much stress/anxiety is this confusion causing you?”; “How much is your child’s situation affecting other relationships in your life?”; and “How well have you found ways to take care of yourself?”. An additional post-program questionnaire item asked parents, “How helpful was the program for you?” in which a 1 indicated the least helpful and a 10 indicated the most helpful. At the end of the 8-week session, the Creating Bonds facilitator instructed parents that they would be receiving an email with an optional survey to complete about the program within the next week. Surveys did not collect any demographic or identifying information, which did not allow us to evaluate individual-level improvements before and after participation because responses could not be linked. Thus, only group-level data were collected before and after the intervention.

#### 2.3.3. Program Feedback

The pre-program questionnaire contained one qualitative item that asked parents, “What topics would you like to discuss during the series?” The post-program questionnaire contained 3 qualitative items that asked parents, “How was the program experience for you?”, “What did you learn from or benefit from in this group?” and “Do you have any recommendations or suggestions for future groups?” Parents were not limited in the length of their qualitative responses.

### 2.4. Analyses

Qualitative and quantitative data were collected in parallel and analyzed separately, with the qualitative data providing additional depth to the understanding of parents’ experiences. Qualitative and quantitative data were intentionally integrated to provide complementary and corroborating evidence regarding parents’ experiences before and after participation in Creating Bonds. Although the same parents participated in pre- and post-questionnaires, only group-level data could be analyzed due to lack of identifying information associated with individual responses. Neither questionnaire collected identifying information, so pre- and post-questionnaires could not be linked. For quantitative analyses, means and standard deviations were obtained for continuous data, and categorical items were described using frequency statistics. For continuous data, independent samples *t*-tests were used to evaluate pre/post group-level differences to quantitative items. Significance levels were set at *p* < 0.05. Data were checked for outliers, and no outliers were found. Quantitative analyses were conducted using SPSS Statistical Software, Version 29 (IBM Corp, Armonk, NY, USA).

Qualitative findings were used to contextualize and deepen interpretation of quantitative outcomes before and after participation in Creating Bonds. Qualitative data were analyzed using an inductive thematic analysis approach [35]. Inductive thematic analysis enables researchers to identify themes from the data rather than applying a predefined framework or theory. The inductive thematic analysis was conducted in three steps: (1) immersion in the topic through a literature review, (2) identification of themes, and (3) coding of themes. Initial thematic analysis was conducted independently by one research team member and confirmed by a second member using NVivo, Version 15 (QSR International, Burlington, MA, USA). Coding was conducted in a stepwise fashion to facilitate iterative revision and finalization of a coding scheme.

## 3. Results

### 3.1. Quantitative Results

#### 3.1.1. Feasibility

Out of the 80 questionnaires administered to 40 parents, a total of 69 pre- and post-program surveys were completed, including 32 pre-program questionnaires (80% completion rate) and 37 post-program questionnaires (93% completion rate) from 6 distinct Creating Bonds cohorts that occurred between March 2022–May 2025, with 6–7 parents per cohort. Parents joined from all regions of the United States and Canada, as well as from European countries, including England and Ireland. On average, parents attended 7 (*M* = 6.95, *SD* = 1.22, range = 3–8) of the 8 total Creating Bonds sessions. Only 9 (24.32%) parents attended fewer than 7 of the 8 sessions. Fifteen (40.54%) parents attended all 8 sessions, and thirteen (35.14%) attended 7 of the 8 sessions.

#### 3.1.2. Acceptability and Perceived Effectiveness

Parents rated the program an average of 8.58 (*SD* = 1.91, range 2–10) out of 10 in terms of helpfulness. Consistent with our hypothesis, results demonstrated that the Creating Bonds program facilitated relationships between parents of children with chronic pain. Prior to joining the Creating Bonds series, the majority (*N *= 27, 84.38%) of parents did not know other parents who had children with chronic pain, and only 5 (13.51%) parents said they knew other parents of children with chronic pain before participating. After the series, 100% of participants responded that they knew another parent who had a child with chronic pain, *t*(67) = −13.93, 95% CI [−0.96, −0.72], *p* < 0.001.

Also consistent with our hypothesis, levels of understanding of pediatric chronic pain and self-care abilities significantly increased, while confusion, stress, anxiety, and relationship conflict significantly decreased. Participants’ levels of understanding pediatric chronic pain significantly improved, *t*(67) = −5.18, 95% CI [−3.35, −1.49], *p* < 0.001. Prior to the series, participants rated their average level of understanding as 5.50 out of 10, which increased to a level of understanding of 7.92 out of 10 after participating in Creating Bonds. Participants rated their level of confusion about how to parent differently as 6.69 out of 10 before the intervention, and levels of confusion significantly decreased to 5.24 out of 10 after the series, *t*(67) = 2.23, 95% CI [0.15, 2.74], *p* = 0.029. In terms of stress and anxiety levels, participants rated their stress and anxiety an average of 7.72 out of 10 before starting the program. Stress and anxiety levels significantly decreased after the Creating Bonds program to an average of 6.43 out of 10, *t*(67) = 2.06, 95% CI [0.04, 2.53], *p* = 0.044. When asked how much their child’s situation affected other relationships, participants responded with a mean rating of 7.41 out of 10. After Creating Bonds, the mean rating of how much other relationships were affected significantly decreased to 6.16 out of 10, *t*(67) = 2.13, 95% CI [0.08, 2.41], *p* = 0.037. Increases in self-care abilities following program participation were also notable. Prior to beginning Creating Bonds, participants rated their average ability to take care of themselves as 4.81 out of 10. After participation, parents’ ratings of their abilities to care for themselves significantly increased to 6.00 out of 10, *t*(67) = −2.16, 95% CI [−0.96, −0.72], *p* = 0.034).

Despite several significant findings, self-reported levels of isolation only moderately decreased and did not achieve statistical significance. Participants rated their pre-program levels of isolation 6.84 out of 10, which slightly decreased to a mean level of 6.24 out of 10 after participation in Creating Bonds, *t*(67) = 0.97, 95% CI [−0.63, 1.83], *p* = 0.335. See Table 3 for mean group ratings across participants before and after Creating Bonds participation.

### 3.2. Qualitative Results

#### 3.2.1. Acceptability

Qualitative findings provided complementary support and greater detail for quantitative results before and after participation in Creating Bonds. When asked what participants were hoping to get out of the Creating Bonds program, qualitative analysis using an inductive thematic approach resulted in identification of three themes: (1) Knowledge about biopsychosocial pain management, (2) Solutions for parenting challenges and balancing family dynamics, and (3) Supporting child identity and social connections. Within the knowledge of the biopsychosocial pain management theme, participants described how they were seeking information about the latest evidence-based medical and psychological therapies for pediatric pain and common comorbidities. The second theme of solutions for parenting challenges and family dynamics highlighted participants’ desires to learn directly applicable parenting strategies that would not reinforce negative pain-related behaviors but supported and encouraged the child with pain, as well as how to encourage independent management to promote optimal functioning for the entire family. In relation to the theme of supporting child identity and social connections, participants expressed interest in encouraging social connections with peers to reduce isolation and ease vital socioemotional and academic transitions for children with pain.

Qualitative analysis using an inductive thematic analysis of post-program participant reflections revealed five overarching themes that highlight both the practical and emotional benefits of participating in the Creating Bonds series, including: (1) Practical parenting strategies and skills, (2) Validation and normalization of the pain experience, (3) Community connection, (4) Emotional relief and growth, and (5) Pain education. See themes, definitions, and example quotes in Table 4.

#### 3.2.2. Practical Parenting Strategies and Skills

In terms of practical parenting strategies and skills, participants reported gaining concrete strategies to better navigate the challenges of raising a child with chronic pain. They described learning “what to say and not to say in various situations,” receiving “non-intuitive parenting tips,” and developing “simple strategies and mindset shifts” that made a “huge difference” in daily interactions, relationships, and ability to care for themselves and their children. Tools such as the “five pillars” of health (sleep, exercise, nutrition, social connection, and stress management), mindfulness, and meditation were highlighted as valuable resources. Participants appreciated specific examples that could be directly applied at home, including important reminders to “not ask the child about their pain level” and encouragement that “Normal functioning precedes reduction in pain lower expectations.”

#### 3.2.3. Validation and Normalization of the Pain Experience

A central perceived benefit of the Creating Bonds program was participants’ reassurance that they were not alone in their challenges. This fostered emotional comfort and shifted parents’ sense of burden from individual to shared. Parents repeatedly emphasized the value of “hearing about other parents’ struggles and their methods,” and “knowing there are other parents out there searching for the same answers.” For some, this addressed long-standing feelings of isolation, as one parent shared: “It was good to hear I’m not the only one facing this”. Recognizing that others faced similar challenges validated and normalized their experiences.

#### 3.2.4. Community Connection

Beyond validation, the Creating Bonds group fostered a sense of connection and belonging within a shared community, which strengthened parents’ resilience. Several participants highlighted the importance of vulnerability and flexibility within the group, and one participant expressed appreciation for the “Super Mamas” community that emerged from the program. The sense of shared experience created a foundation for ongoing support, with participants noting “an immediate connection for additional support.”

#### 3.2.5. Emotional Relief and Growth

Alongside practical skills and a sense of community, participants described emotional benefits such as reassurance, encouragement, and mindset shifts. Group participation helped one parent “learn the necessity of taking care of myself so that I can better take care of my family.” Participants emphasized that the group eased the “anxious energy at home” and helped them adopt a more patient and compassionate approach to parenting. One participant summarized this shift as learning to “be more patient, lower my expectations, and celebrate the positive wins.” These changes fostered both relief and a sense of growth in coping with the demands of parenting a child with chronic pain.

#### 3.2.6. Pain Education

Finally, participants appreciated learning pain management strategies based on scientific evidence. The effectiveness of the program was often attributed to the guidance of the group facilitator, who was frequently praised by participants as “exceptional,” “smart and helpful,” and “lovely to listen to and be guided by.” The program structure and facilitation were viewed by participants as essential to creating a safe, supportive, and educational environment. Participants expressed trust in the pain education, with one stating: “The qualifications of the facilitator were evident, and I could trust her recommendations.” Collectively, these themes capture the dual role of the group in providing tangible parenting tools alongside critical psychosocial support. Table 4 provides themes, definitions, and parental quotes, which illustrate direct parental experiences of each theme.

#### 3.2.7. Qualitative Program Feedback

In terms of suggestions for improving the Creating Bonds program, multiple (*n* = 10) participants recommended increasing the program length to 12 weeks to allow more in-depth exploration of content and facilitate deeper connections among participants. A participant stated, “I feel like we just scratched the surface,” and another added, “We had just gotten to know each other when it was time for the program to end.” One parent expressed interest in receiving electronic PowerPoint slides and handouts that could be referenced after the program concluded. Several (*n* = 12) shared that they did not have any suggestions for improvement and would keep the program as designed. Table 5, which displays condensed themes, illustrates how pre-program expectations aligned with experiences participants shared after the Creating Bonds program concluded.

## 4. Discussion

This study contributes to the growing body of literature demonstrating that having a child with chronic pain exerts a significant impact on the broader family system. As a real-world quality improvement program evaluation involving parents actively seeking support, the findings provide ecologically valid insights into the experiences of families managing pediatric pain. Although demographic characteristics of participating parents were not collected, the absence of strict inclusion or exclusion criteria enhances the generalizability of the findings across diverse populations. The program’s free and easily accessible format underscores its potential scalability and broad reach among parents and families who may otherwise face barriers to care. Importantly, this evaluation demonstrated the strong feasibility of the Creating Bonds program, which was reflected by high engagement and attendance rates, with the majority of participants (75.7%) attending at least seven of eight sessions. Moreover, the program showed high acceptability and perceived effectiveness, evidenced by significant improvements in parent-reported outcomes related to coping, understanding, and family functioning.

Results demonstrated that the 8-week Creating Bonds program significantly improved parents’ levels of understanding of chronic pain, decreased confusion related to parenting strategies, decreased parental stress and anxiety, improved their relationships with others, and significantly improved parents’ abilities to care for themselves. Before participating in the Creating Bonds program, participants were focused on questions, uncertainties, and worries about medical management, parenting, and future transitions. After participating in the program, parents emphasized validation, connection, reassurance, and practical coping strategies—showing a movement from seeking information to gaining confidence, evidence-based knowledge, and community. Overall, parents qualitatively described the program as “educational, validating, and transformative,” stating how it provided both emotional reassurance and concrete skills, which helped families reframe challenges and strengthen coping strategies. Parents came away with practical tools for daily caregiving, but equally important, they experienced emotional relief, community connection, and reduced isolation. Findings suggest that the interplay of shared peer experience, skilled facilitation, and credible expert input created a transformative environment that normalized experiences while equipping parents with strategies to better support both their child and themselves. The dual impact of supportive relationships combined with actionable tools emerged as the primary defining values of the program experience.

Prior to participating in Creating Bonds, parents reported an average self-care ability rating of 4.81 out of 10. Following program participation, ratings significantly increased to 6.00 out of 10, *t*(67) = –2.16, *p* = 0.034, indicating notable improvements in parents’ perceived abilities to care for themselves. Although this increase is statistically significant, the overall mean score suggests that parents of children with chronic pain continue to face substantial challenges engaging in self-care practices amidst the demands of caregiving. Parenting a child with chronic pain often requires sustained emotional, physical, and logistical resources that often limit opportunities for self-care. These findings underscore the importance of integrating targeted self-care components into future interventions for parents of children with chronic pain, as enhancing caregiver well-being may improve family functioning and support the child’s pain management outcomes.

The impact of childhood chronic pain on parents has gained increasing attention as evidence shows how parents with significant emotional distress and poor family functioning can affect health outcomes and a child’s adjustment [11,24]. Parents’ protective and monitoring responses to pain are associated with poorer child outcomes [36]. Healthcare practitioners struggle to meet the needs of parents whose children present for treatment for pediatric chronic pain in various settings by focusing only on the patient with chronic pain and not the parent [37]. Results reinforce how clinicians must include Clinicians who treat Mixed methods approaches to program evaluations that conduct both content and outcomes comparisons for quality improvement purposes, as was done in this study, can provide valuable insights into how to shape and inform the processes of program design and implementation. Within the parent population of children with pediatric pain, it is unknown which parent-focused elements seem consistently desired, accepted, and most helpful to parents in group settings. Results from the present study show that parents desire and appreciate pain education, practical parenting strategies, and group support to reduce feelings of isolation and normalize their experiences. These findings are similar to prior research in which parents demonstrated similar concerns about their child with pain, parenting frustrations, and desire for social support [25].

For families with a child who has a chronic condition, the home environment is often where maladaptive illness behaviors are developed. Parents of children with chronic pain serve as primary models of coping and heavily influence a child’s pain behaviors. Parenting a child with chronic pain can also have a negative impact on many aspects of a parent’s life. It is important for parents to learn to modulate their own distress around their child’s pain to be able to assist their child to effectively self-manage their pain. Supporting and educating parents to effectively cope with their child’s chronic pain and care for themselves is a critical need. In conjunction with gold-standard, multidisciplinary pain care for youth with pain, the Creating Bonds program for parents can help fill the current gap in comprehensive, whole-family pain care.

This is the first study to report on the Creating Bonds parent intervention, and results point to the strengths of the program. Nonetheless, several limitations exist. For the purpose of this quality improvement study, demographic data on parent participants was not collected. It is unknown whether the Creating Bonds program was more or less effective or acceptable based on specific demographic characteristics of participants. Throughout the 8-week series, we were unable to control for whether participants engaged in additional supportive therapies outside of Creating Bonds to increase their knowledge and understanding of how to parent a child with chronic pain. It is possible that additional interventions may have contributed to the positive results after the program. This study did not assess any outcomes among children of parent participants or concurrent participation of youth in other programs offered through Creative Healing for Youth in Pain. Finally, this quality improvement program evaluation consisted of a non-randomized design. Thus, a larger, multi-site RCT study would be valuable to replicate and extend these findings with larger, diverse participant samples. Future studies should also aim to evaluate the impact of parent programs on child outcomes, parent–child interactions, and other domains of parental socioemotional functioning, including anxiety and psychological adjustment.

## 5. Conclusions

Parents provide a major influence in children’s lives that can have a positive or negative effect on a child’s socioemotional and health outcomes. Additionally, parents of youth with chronic pain often experience elevated distress related to the demands of caregiving. The role that parenting behaviors play for families with a child with chronic pain was reviewed. Compared to pre-program scores, post-program results showed that Creating Bonds, an online group intervention for parents of youth with chronic pain, had positive effects on multiple parent-reported qualitative and quantitative outcomes. Taken together, these findings highlight the promise of parent-focused online interventions as a feasible and helpful approach to supporting families navigating pediatric chronic pain. Future randomized trials with larger and more diverse participant samples, as well as the use of control groups, may help to compare the intervention to other parent interventions and to determine changes in child health outcomes following parent-reported improvements.

## Figures and Tables

**Table 1:** Mindfulness Exercises in Creating Bonds.

Week	Mindfulness Exercises
Week 1	Group introductions
Week 2	Group introductions and education on acute versus chronic pain
Week 3	Introduction to Mindfulness and accessing through senses
Week 4	Mindfulness technique of letting thoughts float away
Week 5	Teach 5 different breathing techniques so parents can continue what they connected with in their home practices
Week 6	Safe place meditation
Week 7	Self-compassion meditation
Week 8	Nurturing figures meditation for self-care

**Table 2:** Common Topics and Themes Covered in Creating Bonds.

Topics
Lecture on acute versus chronic pain and how chronic pain develops Review the 5 pillars of mental and physical health Encourage self-care, such as quick stretching, deep breaths, talking to a friend, exercising, or a creative hobby Teach Rules of Chronic Pain (e.g., not asking how the child is feeling, not pointing out when the child seems better, acknowledge when child is in pain) Guidance on feeling helpless when the child is in pain Discuss impact of pain on other kids/siblings and the family Discuss school reintegration Encourage guiding the child to function in normal activities Review how parents can balance when to push and when to empathize or coddle regarding school, chores, and other expectations Provide guidance on how to deal with conflicting recommendations from various physicians Provide guidance on how to support the child with feelings of isolation Dealing with marital conflict stemming from parenting the child in pain How to work through the child regressing when they were making improvements Teach how to determine if the child is faking or exaggerating symptoms How to reconcile that the child seems fine when doing something they enjoy, but say school is too difficult to handle with pain Approaching a child’s fears about recovery hindering recovery How to deal with the reactions of teachers, parents, and community members to the child’s pain Support parents grappling with the concept of the child’s increasing functioning before the pain decreases for recovery Approach concerns that the child feels guilty about the financial, emotional and overall impact his/her pain is having on the family Support parental coping with traumatic feelings from the medical system (doctors not believing your child, saying insensitive things, talking to the parent instead of to the child, saying it is all in their head, etc.) How to deal with extended family and friends not understanding what the child is experiencing

**Table 3:** Mean group-level pre/post ratings to quantitative survey items.

Item	Pre *N* = 32 *M* (*SD*)	Post *N* = 37 *M* (*SD*)	Range	*p*-Value
Do you know other parents who have children with chronic pain?	5 (84.4%)	37 (100.0%)	Y/N	<0.001
Yes				
What is your level of understanding of pediatric chronic pain?	5.50 (2.40)	7.92 (1.42)	1–10	<0.001
How isolated do you currently feel because of having a child with pain?	6.84 (2.40)	6.24 (2.69)	1–10	0.335
How confused do you feel about how to parent differently than you usually would, such as when to push and when to coddle?	6.69 (2.61)	5.24 (2.75)	0–10	0.029
How much stress/anxiety is this confusion causing you?	7.72 (2.23)	6.43 (2.86)	1–10	0.044
How much is your child’s situation affecting other relationships in your life?	7.41 (2.08)	6.16 (2.68)	1–10	0.037
How well have you found ways to take care of yourself?	4.81 (2.32)	6.00 (2.24)	1–10	0.034

**Table 4:** Examples of Parental Quotes by Theme.

Theme	Definition	Example Quotes
Practical Parenting Strategies and Skills	Concrete strategies and skills helped parents adjust their approaches and reduced uncertainty.	“I learned not to ask about my child’s pain and that increased functioning will help retrain the brain.”
Community Connection	Shared experiences normalized parents’ struggles and reduced isolation.	“Knowing I am not alone is helpful in itself.” “We could relate to one another.”
Validation and Normalization of the Pain Experience	Parents felt validated in their struggles, parenting, and in recognizing their children’s pain experiences.	“Validation of my parenting approach.” “Support of other parents in same spot.”
Emotional Relief and Growth	Parents valued being reminded to prioritize themselves and the emotional side of pain, which helped reduce stress and promote resilience.	“Knowing I’m doing the right thing helps relax the anxious energy at home.”
Pain Education	New knowledge helped parents better understand chronic pain and what their children were going through.	“I learned how to talk or not talk about pain in a scientifically informed way, and what is actually going on in the body.”

**Table 5:** Program Expectations and Experiences by Condensed Themes.

Themes	Pre-Program Expectations	Post-Program Experiences
Practical Parenting Strategies and Pain Education	Understanding treatment options, when to seek new providers Comorbidities (anxiety, depression, GI diagnoses) Balancing medical and psychological care	Gained education on biopsychosocial approach to pain management Therapy recommendations from other parents Relief in learning that functioning precedes pain reduction
Emotional Relief and Growth	How to balance support vs. independence Managing discipline, scheduling, motivation Coping with future uncertainty Impact on siblings and family dynamics Preserving hope and joy	Gained concrete parenting strategies (e.g., not asking about pain, focusing on functioning) Validated by shared medical/psychological challenges Learned importance of self-care to support family Learned value of mindfulness, relaxation, self-care Reassurance that they are “not alone” in struggles Built resilience through group support and shared experiences
Community Connection and Validation and Normalization of the Pain Experience	Helping child form identity beyond pain Navigating school, teacher relationships Transition to college/young adulthood Finding social connections, reducing isolation	Connection with peers and other parents Felt supported in navigating adolescence and identity concerns Gained hope seeing others with similar struggles Reported relief and reassurance in shared experiences and group cohesion

## Data Availability

The raw data supporting the conclusions of this article will be made available by the authors upon request. Data are not publicly available due to privacy and ethical reasons.

## Abbreviation

The following abbreviation is used in this manuscript:

CHYP	Creative Healing for Youth in Pain
